# Design and Analysis of MEMS Based PVDF Ultrasonic Transducers for Vascular Imaging

**DOI:** 10.3390/s100908740

**Published:** 2010-09-21

**Authors:** Chaitanya Chandrana, James Talman, Tao Pan, Shuvo Roy, Aaron Fleischman

**Affiliations:** 1 Department of BioMedical Engineering, Cleveland Clinic, Cleveland, OH, USA; E-Mails: Chaitanya.Chandrana@sri.utoronto.ca (C.C.); Taopan@gmail.com (T.P.); 2 Department of Chemical and Biomedical Engineering, Cleveland State Univeristy, Cleveland, OH, USA; 3 Offboard Countermeasures Branch, United States Naval Research Laboratory, Wahington, DC, USA; E-Mail: Jim.Talman@nrl.navy.mil (J.T.); 4 Department of Bioenginering & Therapeutic Sciences, University of California, San Francisco, CA, USA; E-Mail: Shuvo.Roy@ucsf.edu (S.R.)

**Keywords:** ultrasound transducer, high-frequency ultrasound imaging, parasitic capacitance

## Abstract

Polyvinilidene fluoride (PVDF) single-element transducers for high-frequency (>30 MHz) ultrasound imaging applications have been developed using MEMS (Micro-electro-Mechanical Systems) compatible techniques. Performance of these transducers has been investigated by analyzing the sources and effects of on-chip parasitic capacitances on the insertion-loss of the transducers. Modeling and experimental studies showed that on-chip parasitic capacitances degraded the performance of the transducers and an improved method of fabrication was suggested and new devices were built. New devices developed with minimal parasitic effects were shown to improve the performance significantly. A 1-mm aperture PVDF device developed with minimal parasitic effects has resulted in a reduction of insertion loss of 21 dB compared with devices fabricated using a previous method.

## 1. Introduction

High-frequency (>30 MHz) ultrasound imaging, with its microscopic resolution, has opened up new areas of medical study in the fields of ophthalmology, dermatology, and intravascular imaging (IVUS) [1,2]. In these imaging fields, image resolution is primarily determined by the properties of the transducer. Various transducer materials have been investigated for use in high-frequency imaging. These include piezoelectric materials such as lead zirconate titanate (PZT) [3], polyvinilidene fluoride (PVDF) [4], as well as silicon nitride, which has been used in capacitive micromachined ultrasonic transducers (CMUT) [5]. PZT is usually used in medical imaging devices because of its higher sensitivity. However, PVDF and its copolymers (PVDF TrFE) offer unique advantages over other materials including broadband width, mechanical flexibility, better acoustic impedance match to tissue, and lower cost [3]. As such, broad bandwidth, focused transducers with desirable properties for high-resolution medical ultrasound have been developed using polymers [4]. However, miniaturized polymer transducers have high electrical impedance compared to PZT [5], which causes an electrical impedance mismatch to the typical 50 Ω load of electronic instruments, which comprise the signal/image processing console [6]. Impedance-matched networks can match the high impedance to the 50 Ω receiver, but with a poorer impulse response [7]. High input impedance amplifiers in close physical proximity to the transducer have been used to prevent reduced SNR due to loading of the transducer by the corresponding signal/image processing console [8]. This approach can be implemented by integrating amplifier components with PVDF film, which along with its copolymers, are compatible with integrated circuit (IC) fabrication processes and are amenable with MEMS fabrication strategies [9]. Several groups have integrated polymer ultrasonic transducers with electronics [9,10]. Our group has demonstrated the fabrication of miniature, high-frequency, focused PVDF transducers [11], where techniques compatible with CMOS microelectronics and MEMS fabrication processes were demonstrated to produce ultrasonic transducers capable of being integrated onto a monolithic chip.

In this paper, the effect of on-chip parasitic capacitance on the performance of MEMS-based ultrasound transducers was investigated. Focused PVDF transducers using MEMS compatible protocols were developed and the on-chip parasitic capacitances for these devices were identified and measured. An alternate transducer model with minimal parasitic capacitance was proposed and a prototype device was developed and tested. The new minimal parasitic devices showed similar imaging resolutions as the previous devices the new devices with 1-mm diameter showed ∼21 dB improvement in insertion loss.

## 2. Device Development

**Figure 1.**
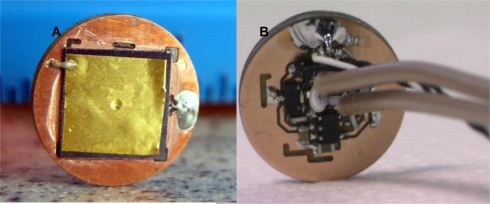
**(A)** Photograph of a focused 1-mm transducer on a 1-cm^2^ silicon chip attached to a custom PC board. **(B)** Amplifier electronics along with input and output cables placed on the PC board that would be placed at the back of the transducer.

Focused PVDF TrFE transducers were developed for minimally invasive procedures using pressure deflection and micromachining techniques [12]. A piezoelectric PVDF film (with one side coated with Cr/Au for electrical contact) is placed on the silicon substrate with a micromachined circular aperture in the center. The Si substrate has a layer of SiO_2_ with a thickness of 1.5 7 μm along the inner circumference and on the top surface of the silicon substrate. The PVDF film with the substrate is firmly inserted onto a jig such that the non-metalized side of the film is in contact with the silicon. Pressure is applied on the PVDF TrFE film stretched over the micromachined aperture in the silicon substrate by sending air through the small nozzle attached to the bottom of the jig. The air pressure deflects the polymer film such that it forms a spherical section. The air pressure injected onto the film can be manually controlled thereby controlling the deflection of the film. A silver epoxy is then applied to the back of the opening to preserve the shape of the film, and also to provide electrical contact to the non-metalized PVDF surface. After the epoxy is cured, the air pressure is removed. Spherical shape of the transducer is retained and a focused transducer is formed. Ultrasonic transducers with various focal numbers can be fabricated using the membrane deflection technique by simply changing the air pressure during fabrication. These transducers are later connected to a high-impedance pre-amplifier mounted on a custom PC board [12]. The transducer and amplifier components are shown in Figure 1.

## 3. Identification of On-Chip Parasitic Capacitances

**Figure 2.**
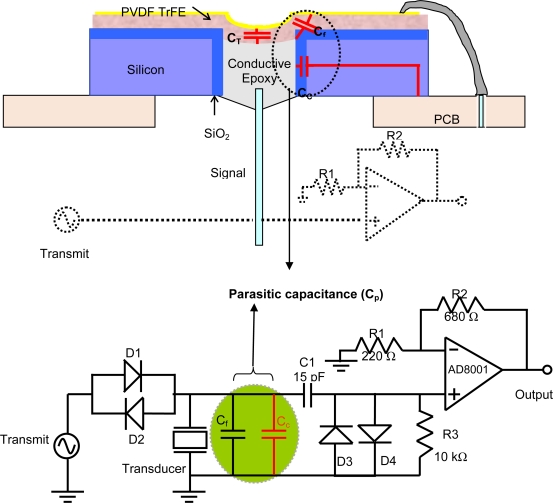
Cross-sectional diagram and schematic of the device showing the inherent and parasitic capacitances associated with the device.

A cross-sectional schematic of the transducer attached to a printed circuit board is shown in Figure 2. It can be seen that there are three capacitors associated with this device:

1. Capacitance from PVDF polymer film itself (labeled *C_T_* in Figure 2). This is the inherent capacitance from the film and will always be present.
2. The capacitance due to 1.5-μm layer of SiO_2_ along the inner circumference of the Silicon chip. The SiO_2_ layer, which isolates the conductive epoxy from the conductive silicon wafer, acts as a cylindrical capacitor (*C_C_*) between the conductive epoxy used as a backside electrode and silicon substrate.
3. The capacitance due to fringing fields in the dielectric of the substrate (*C_f_*). *C_f_* is considered much smaller than *C_C_*.

The total parasitic capacitance, denoted Cp, is given by the sum of Cc and C_f_. *C_P_* forms a parallel impedance to the output and could significantly reduce the input to the preamplifier, thereby degrading the performance of the transducer.

## 4. Analysis and Modeling

**Figure 3.**
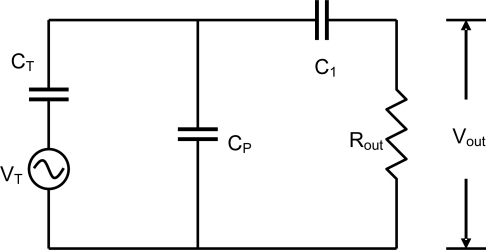
Circuit model to simulate the effect of parasitic capacitance.

A circuit model was developed to simulate the effect of parasitic capacitance and is shown in Figure 3. The model considers the electrical circuit during receive mode, when the transducer acts as a source. The source voltage (*V_T_*) is proportional to the voltage generated by the transducer during receive mode. Transducer capacitance (*C_T_*), Parasitic capacitance (*C_P_*), Coupling capacitance (*C_1_*), and output resistance (R_out_) are as denoted in the schematic. R_out_ provides a path for DC bias current to the amplifier. In the receive mode, the transducer acts as a voltage source and generates an electric signal. This signal is attenuated by the parasitic capacitance in parallel with the series combination of C*_1_* and R_out_. The effect of parasitic capacitance was evaluated using the transfer function given below.

(1) $$\frac{V_{\mathrm{out}}}{V_{T}}\;=\;\frac{1}{1\;+\;\frac{1}{2\pi \;\cdot \;f\;\cdot \;R_{\mathrm{out}}}\left(\frac{1}{C_{1}}\;+\;\frac{1}{C_{T}}\right)\;+\;\left(1\;+\;\frac{1}{2\pi \;\cdot \;f\;\cdot \;R_{\mathrm{out}}C_{1}}\right)\frac{C_{p}}{C_{T}}}$$

In Equation (1), all variables are constant except *C_P_*. *C_T_* is the inherent capacitance of the transducer. Typical *C_P_* and *C_T_* values for a 1-mm transducer are 25 pF and 1.8 pF respectively. C*_1_* is the coupling capacitance between the transducer and the amplifier, and its value is 15 pF by design. *R_out_* is the input impedance of the amplifier (10 K) and is constant.

## 5. Device with Minimal On-Chip Parasitics

**Figure 4.**
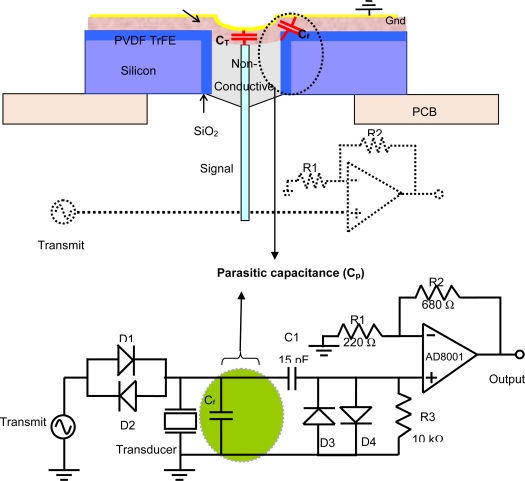
Cross-sectional and schematic of the suggested transducer model with minimal parasitic capacitance.

This section presents an approach to develop a minimal parasitic device. Schematic of the suggested model is shown in Figure 4. This model consists of a non-conductive mechanical backing and the electrodes are connected to the top and bottom of the PVDF film. This configuration eliminates the parasitic capacitance associated with silicon substrate and would be the preferred configuration for an integrated transducer.

For testing the suggested model, prototype devices that would eliminate the parasitic capacitance were fabricated using polycarbonate substrate instead of silicon. Polycarbonate was chosen to eliminate the cylindrical capacitance C*_c._* which is the dominant contribution to the parasitic capacitance. Although polycarbonate is not a semiconductor and therefore cannot be used to create a preamplifier for an integrated transducer in a MEMS fabrication process, it is employed to clearly demonstrate the effects of removing the parasitic capacitance. This model is electrically identical to the suggested model with non-conductive epoxy. The only difference between the two devices is the fringing capacitance, which can be ignored. The dielectric constant of Polycarbonate (2.9) also is much lower than that of Si (11.6) and SiO_2_ (4.5) and hence the fringing capacitance of the polycarbonate devices would be smaller than the final devices. Prototype transducers were fabricated on polycarbonate substrate using the same pressure-deflection technique used to create the transducers on a silicon substrate.

## 6. Parasitic Capacitance Measurement

**Figure 5.**
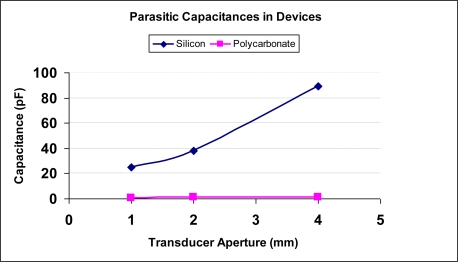
Measurements of parasitic capacitance with silicon and polycarbonate. It can be seen that silicon devices have higher capacitances compared to polycarbonate devices

Silicon (parasitic) and polycarbonate (minimal parasitic) transducers with various apertures (1-mm, 2-mm and 4-mm) were fabricated using techniques described in previous sections. The substrate capacitances were measured using an impedance analyzer. The impedance analyzer was first calibrated to eliminate for the parasitic contribution from the cables connected to the active transducer element via the open, short and 50Ω load cables. Next, the device with its cable was connected to the analyzer to measure its impedance (transducer + parasitic capacitances). In order to single out the parasitic capacitance from the device measurements, it was necessary to isolate the transducer capacitance. This was achieved by carefully peeling off the section of the PVDF film that strictly contributes to the transducer capacitance (spherical section at the center of the chip). The remaining film was left unpeeled to make electric contact. Impedance measurements from such modified devices yielded the required parasitic capacitance. The measured parasitic capacitance values for silicon devices were significantly higher than the corresponding measurements from polycarbonate devices. Figure 5 shows the parasitic capacitance measurements for silicon and polycarbonate devices with various apertures. For 1, 2 and 4 mm aperture transducers, the parasitic capacitance was measured to be 25, 38.2, and 89.3 pF for silicon transducers and 0.71, 0.95, and 1.1 pF for polycarbonate transducers respectively.

## 7. Analysis of On-Chip Parasitic effect

**Figure 6.**
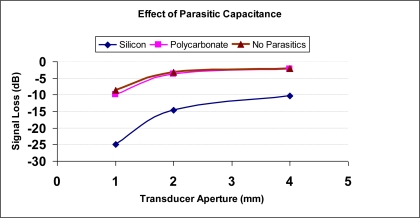
Effect of parasitic capacitance on signal loss for various aperture transducers. Polycarbonate devices show lower signal loss and comparable to losses from devices having no parasitic capacitances.

The effect of parasitic capacitance on the output of the transducer was analysed using the developed model (Figure 3). The model was analyzed using electrical simulation software, PSPICE (Cadence, San Jose, CA). The output voltage was simulated for three cases—silicon devices (with parasitics), polycarbonate devices (minimal parasitic) and for devices with no parasitics. For the simulation measurements, a 40-MHz monocycle pulse with peak amplitude of 50 mVpp was used as the driving voltage. The amplitude is significantly smaller than the excitation voltage as only the propagation in receive mode was considered and this amplitude might closely represent the signal from the transducer in the receive mode. The same voltage signal was considered for all the transducers just to be consistent and to make a comparative assessment of the final output. Transducer capacitance *C_T_* values of 1.8, 11.7 and 53.3 pF were used for 1, 2 and 4 mm devices respectively. Parasitic capacitance *C_P_* values obtained for silicon and polycarbonate devices (in previous section) were used in this model. For the device with no parasitics, *C_P_* was removed from the model. The output voltages for the various devices with different parasitic capacitances were modeled. Figure 6 shows the voltage loss due to parasitic capacitance for 1-mm, 2-mm, and 4-mm transducers, all at 40 MHz. The results show that polycarbonate-based transducers have lower voltage losses (15 dB, 11 dB, and 8 dB) compared with transducers fabricated on a silicon substrate. The polycarbonate transducers, with minimal parasitics showed almost identical signal losses as the transducers with no parasitics.

## 8. Pulse-Echo Response of Parasitic and Minimal Parasitic Devices

**Figure 7.**
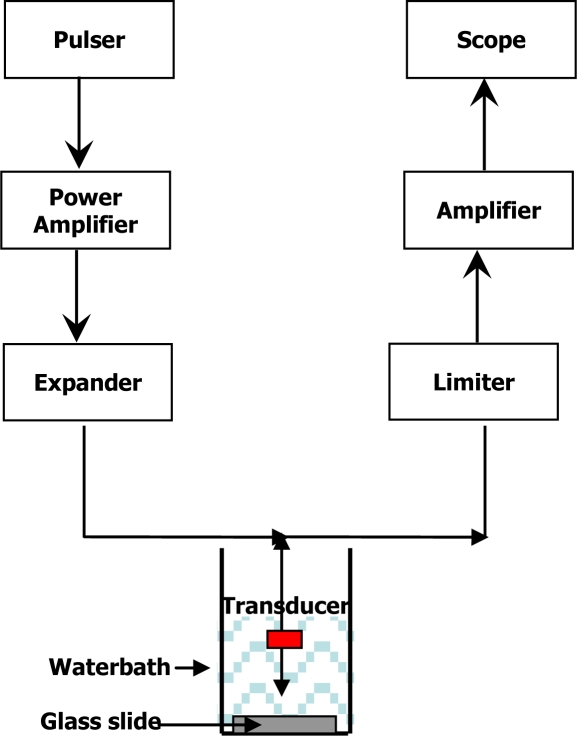
Experimental imaging apparatus.

**Figure 8.**
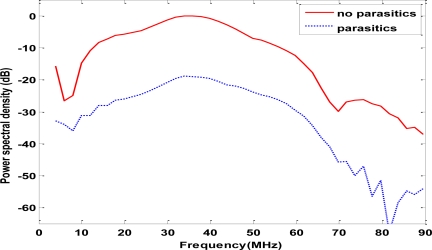
Power spectral densities of polycarbonate (no parasitics) showing signal loss compared to and Silicon (parasitic) transducers. Both of them have nearly identical center-frequencies and bandwidths.

Pulse-echo responses from the 1-mm parasitic (silicon) and minimal-parasitic (polycarbonate) transducers were obtained by exciting the transducers with 40-MHz, 50 V_pp_ pulse at a 2 KHz repetition rate. The experimental set up used for obtaining pulse-echo responses is given in Figure 7. The transducer is placed in waterbath and reflections from the glass plate at the bottom of the waterbath are recorded. The pulse-echo response is characterized by the maximum echo along the transducer axis. Power spectral densities (PSD) were obtained by taking Fourier transform of the pulse-echo and are shown in Figure 8. The PSDs were normalized to the peak of the minimal parasitic signal. The center frequencies of silicon and polycarbonate transducers were measured to be 36, 34 respectively and the—6dB bandwidths were 86 and 84 %. The difference can be considered negligible considering the variations in film thickness during the film manufacture. These results lead us to believe that the lateral and axial imaging resolutions of the transducers are the same.

## 9. Insetion-Loss Measurements

In order to obtain a realistic effect of the parasitic capacitance, insertion loss was compared between silicon and polycarbonate transducers with various aperture sizes with and without preamplifier. For silicon transducers aperture sizes used were 0.75 mm, 1 mm and 2 mm aperture sizes. Polycarbonate transducers had aperture sizes of 0.8 mm, 1 mm and 2 mm. The smallest transducers do not have the exact same apertures for silicon and polycarbonate transducers, as it was challenging to fabricate precise holes without the MEMS technology. Nevertheless, the aperture sizes were close enough to give an idea about the parasitic effects.

The insertion losses were calculated from the pulse-echo measurements as

(2) $$\text{Insertion}\;\text{loss}\;(\text{dB})\;=\;20\;•\;{\text{log}}_{10}\frac{{\mathrm{Voltage}}_{\mathrm{pulse-echo}}}{{\mathrm{Voltage}}_{\mathrm{input}}}$$

**Figure 9.**
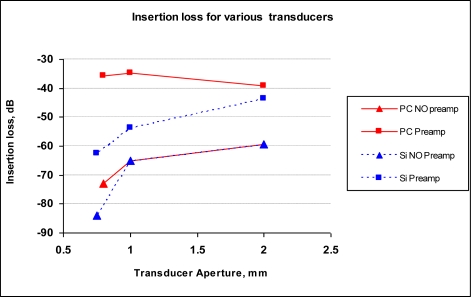
Insertion loss measurements of Silicon and Polycarbonate transducers with and without preamplifiers

The results indicate that polycarbonate transducers with preamplifier exhibit lower insertion losses compared to the silicon transducers with preamplifier, confirming the significant effect of parasitic capacitance on the transducer performance. The results were shown in Figure 9. For amplified transducers with apertures of 1-mm and 2-mm, polycarbonate-based devices showed ∼21 dB and ∼5dB improvement, respectively compared to silicon-based devices. Better improvement in the response from the smaller transducer (lower capacitance and higher impedance) demonstrates the importance of impedance isolation provided by the amplifier.

## 10. Conclusion

High frequency PVDF transducers have been fabricated using MEMS compatible process. The impact of on-chip parasitic capacitance on the performance of miniaturized ultrasonic transducers has been studied via modeling and experiments. Modeling and experimental results showed that on-chip parasitic capacitances significantly degrade the performance of the transducer. A new model with minimal parasitic capacitance was developed. The new model improved the insertion loss of the 1-mm transducer by ∼21 dB while preserving the imaging resolutions. The work shows that the preferred structure for an integrated transducer is to use a non-conductive epoxy for mechanical backing of the transducer and a thin film electrode for backside contact as part of the integrated process for the transducer.

The desired outcome of this research is a single integrated MEMS PVDF transducer chip, combining a high input impedance preamplifier and focused transducer. This work shows an approach to building integrated PVDF transducers with minimal parasitics that could be widely used in clinical IVUS applications.
